# Use of Functionally Graded Material to Decrease Maximum Temperature of a Coating–Substrate System

**DOI:** 10.3390/ma16062265

**Published:** 2023-03-11

**Authors:** Aleksander Yevtushenko, Katarzyna Topczewska, Przemysław Zamojski

**Affiliations:** Faculty of Mechanical Engineering, Bialystok University of Technology (BUT), 45C Wiejska Street, 15-351 Bialystok, Poland

**Keywords:** functionally graded materials, thermal barrier coating, frictional heating, temperature

## Abstract

A mathematical model for determining the temperature distribution in the system consisting of a coating deposited on the surface of substrate was proposed. The foundation material is homogeneous, while the coating is made of a functionally gradient material (FGM) with thermal conductivity increasing exponentially along the thickness. Heating processes of the outer surface of the coating were considered with a constant and linearly decreasing in time intensity of the heat flux. Such thermal loads are common in thermal problems of friction, particularly regarding frictional heating during braking. An exact (in quadrature) solution of the corresponding boundary-value problems of parabolic heat conduction was obtained. Asymptotic solutions to these problems were also found for small and large values of the Fourier number. Calculations were performed for a coating made of two-component FGM ZrO_2_—Ti-6Al-4V, applied on a cast iron substrate. In order to explain the effect of FGM on temperature, corresponding analysis was carried out for the coating made of a homogeneous (ZrO_2_) material.

## 1. Introduction

Development of protective coatings on the frictional components has revolutionized tribological systems in many industrial applications [1]. Properly selected and produced coatings allow to improve reliability and performance of the systems, by enhancing the tribological properties on the contact surfaces, which is essential in the case of friction cooperative elements. The protective layers called thermal barrier coatings (TBCs) are deposited on the outer surfaces of frictional elements of the heavy-loaded tribosystems, which operate under elevated temperature, to increase the components’ durability and alleviate the damage that arises from contact in such extreme conditions. Besides the thermal resistance, the TBCs can protect them from wear and significantly increase the service life of the friction pair elements. In order to get these features, the hard coatings are applied on the relatively soft substrates [2]. Metals and alloys are frequently used as a foundation of the coated elements to maintain structural rigidity and strength. Whereas, ceramic materials are considered as good outer layers for the tribological components, to ensure high hardness, as well as thermal and wear resistance. Miscellaneous particles such as zirconium dioxide (Zr_2_O), aluminum oxide (Al_2_O_3_), silicon carbide (SiC), tungsten carbide (WC), and yttria-stabilized zirconia (YSZ) are employed for coatings of the friction elements in braking systems [3,4,5,6,7,8]. Ceramic intrinsically possess low fracture toughness, so TBCs are susceptible to mechanical or thermal stress-induced failures such as delamination or spallation of the top coating under harsh thermal conditions [9,10,11]. Especially, cracks of the brittle ceramic coating may be initiated in the friction elements of braking systems, when the concentration of subsurface tensile stresses appear due to the frictional heating [12]. Many properties of ceramic TBC are very different from that of metallic substrate, so their direct application may lead to cracking due to the thermal expansion mismatch at the interface of the core and the outer layer [13]. The interface of the coating-substrate system has been proved to be the most critical location for failure [1].

In order to overcome the mentioned problems of conventional ceramic coatings, the concept of functionally graded material (FGM) has been applied to the TBCs, obtaining the functionally graded coatings (FGCs). These coatings are characterized by smooth, graded transition of composition from the outer surface to the inside of the element. This feature tend to reduce stress concentrations resulting from materials mismatch, reinforce the bond cohesion, and significantly increase resilience and fracture strength [14,15,16,17,18,19]. Recently published [20] thermo-mechanical coupled sliding contact analysis of functionally graded coating-substrate structure showed that controlling the gradient parameter of FGC can decrease the residual tensile stress near the friction surface. Additionally, it was found that the functionally graded coating exposed to thermal shock may suffer far less damage than the corresponding homogeneous ceramic cover [21]. The functionality of FGC can be optimized by selecting appropriate gradation of material properties, while an inappropriate gradation can have adverse effects [22]. Therefore, thermal behavior of the friction elements coated with FGCs is an ongoing research topic including heat conduction problems and thermal stress analyses [5,14,20,23,24,25]. The investigation of thermally induced stress is crucial for predicting the failure mechanism such as fracture and crack propagation in the functionally graded materials [26]. Therefore, it is important to develop computational models used to evaluate the thermal response of functionally graded coatings during the design stage of friction couple.

In general, analytical solutions of the sliding contact problems are difficult, or even impossible, to be found for nonhomogeneous materials with finite dimensions [27,28]. As a consequence, the numerical solutions are primarily used for analysis of FGCs, mostly by means of the finite element approximation [5,10,15,18,23]. For instance, solution of the transient one-dimensional heat conduction equation with the arbitrary type of time-dependent boundary conditions has been obtained in [29] employing the central finite difference method. An analysis of the thermo-mechanical coupled problem in functionally graded metal/ceramic plates has been conducted by means of graded finite elements in [23].

The closed-form, exact solutions of the heat conduction and thermoelasticity problems for FGMs are possible to achieve only for a few cases with particular types of boundary conditions, geometry restrictions, and distributions of material properties [23]. The main issue encountered is concerned with modeling continuously varying gradient of material structure. To overcome this problem, most FGM models use the multi-layered approximation approach [2,19,20,22,23,30]. This method relies on a model of graded material heterogeneity by a package of homogeneous layers, which leads to a stepwise change in coating properties along the gradient direction. Two-dimensional frictional contact problem involving rigid stamps and half-plane with functionally graded coating has been considered in [22], introducing a multi-layer method, the gradation of Young’s modulus in coating has been modeled as a piecewise constant function. Using this method, the two-dimensional heat conduction problem for functionally graded circular hollow cylinders subjected to transient thermal boundary conditions with radially dependent properties has been solved in [31]. Another solution of three-dimensional problem of elasticity for functionally graded coated half-space has been achieved in [2].

Our previous research on mathematical modeling of the FGMs friction heating process, presented in articles [24,27,32,33], was based on the semi–infinite body (half–space) model of one or both elements of the friction pair. The main difference between the results presented in this manuscript is the inclusion in the model of the finite thickness of one element—a FGC. It is also important, from the point of view of practical applications, obtaining asymptotic solutions for small and large values of the Fourier number, which do not require numerical integration. It was shown that the form of the obtained exact solution of the problem with uniform heating of the FGM surface of the coating allows for taking into account other than constant time profiles of the heat flux intensity using the Duhamel’s theorem. This is important when modeling the temperature mode of braking systems, when the specific friction power, i.e., also the intensity of the heat flux, significantly changes with the braking time [12].

In this study, a thermal problem of friction was considered for a finite coating made of two-component FGM deposited on the semi-infinite homogeneous substrate, subjected to heat flux with constant and time-dependent intensities. An exponential variation of the thermal conductivity of the FGC along its thickness was adopted in the model. Obtained analytical solutions allow to determine the spatial-temporal distribution of temperature generated due to friction. The effect of FGM application was investigated, by comparing the results calculated for a FGC and for corresponding homogeneous ceramic coating.

## 2. Statement to the Problem

**Figure 1:**
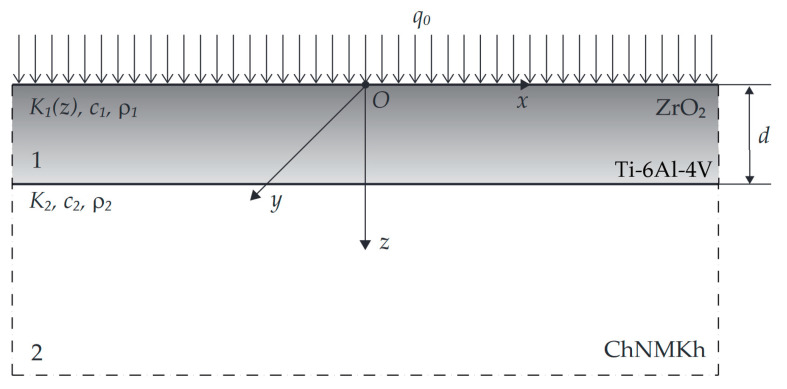
Scheme of heating the coated half-space.

Consider a coated body, with outer layer 0≤z≤d made of two-component FGM deposited on the surface z=d of the homogeneous foundation z≥d (Figure 1). Thermal conductivity of the coating material $K_{1}$ increased exponentially along its thickness [34,35]:

(1) $$K_{1}(z)=K_{1,1}e^{\gamma^{*}z/d},\;0\leq z\leq d,$$

where $\gamma^{*}\geq 0$ is the dimensionless parameter of gradient FGM [36], $K_{1,1}\equiv K_{1}(0)$ and $K_{1,2}\equiv K_{1}(d)$ are thermal conductivity coefficients of the FGM components.

At the initial time moment t=0 the temperature T in the whole semi-infinite region z≥0 is constant $T=T_{0}$. Then, over the time t>0 surface of the coating z=0 is subjected to heating by the heat flux with constant intensity $q_{0}$. Assuming that the thermal contact between coating and foundation is perfect, the transient temperature field $T(z,t)=T_{0}+\Theta (z,t)$, z≥0, t≥0 caused by the heating, can be found from solution to the following boundary-value problem of heat conduction:

(2) $$\frac{\partial }{\partial z}\left[K_{1}(z)\frac{\partial \Theta (z,t)}{\partial z}\right]=\rho_{1}c_{1}\frac{\partial \Theta (z,t)}{\partial t},\;0<z<d,\;t>0,$$

(3) $$K_{2}\frac{\partial^{2}\Theta (z,t)}{\partial z^{2}}=\rho_{2}c_{2}\frac{\partial \Theta (z,t)}{\partial t},\;z>d,\;t>0,$$

(4) $${\left.K_{1}(z)\frac{\partial \Theta (z,t)}{\partial z}\right|}_{z=0^{+}}=-q_{0},\;t>0,$$

(5) $$\Theta (d^{+},t)=\Theta (d^{-},t),\;t>0,$$

(6) $${\left.K_{1}(z)\frac{\partial \Theta (z,t)}{\partial z}\right|}_{z=d^{+}}={\left.K_{2}\frac{\partial \Theta (z,t)}{\partial z}\right|}_{z=d^{-}},\;t>0,$$

(7) Θ(z,t)→0, z→∞, t>0,

(8) Θ(z,0)=0, z≥0,

where $K_{2}$ is the thermal conductivity coefficient of the substrate material; $\rho_{l}$, $c_{l}$ are respectively the density and the specific heat capacities of the materials of coating (l=1) and substrate (l=2).

Introducing the dimensionless variables and parameters:

(9) $$\zeta =\frac{z}{d},\;\tau =\frac{k_{1}t}{d^{2}},\;K^{*}=\frac{K_{2}}{K_{1,1}},\;k^{*}=\frac{k_{2}}{k_{1}},\;\Theta^{*}=\frac{\Theta }{\Lambda },$$

where

(10) $$\Lambda =\frac{q_{0}d}{K_{1,1}},\;k_{1}=\frac{K_{1,1}}{c_{1}\rho_{1}},\;k_{2}=\frac{K_{2}}{c_{2}\rho_{2}},$$

the problems (2)–(8) can be written in the form:

(11) $$\frac{\partial^{2}\Theta^{*}(\zeta ,\tau )}{\partial \zeta^{2}}+\gamma^{*}\frac{\partial \Theta^{*}(\zeta ,\tau )}{\partial \zeta }-e^{-\gamma^{*}\zeta }\frac{\partial \Theta^{*}(\zeta ,\tau )}{\partial \tau }=0,\;0<\zeta <1,\;\tau >0,$$

(12) $$\frac{\partial^{2}\Theta^{*}(\zeta ,\tau )}{\partial \zeta^{2}}-\frac{1}{k^{*}}\frac{\partial \Theta^{*}(\zeta ,\tau )}{\partial \tau }=0,\;\zeta >1,\;\tau >0,$$

(13) $${\left.\frac{\partial \Theta^{*}(\zeta ,\tau )}{\partial \zeta }\right|}_{\zeta =0^{+}}=-1,\;\tau >0,$$

(14) $$\Theta^{*}(1^{+},\tau )=\Theta^{*}(1^{-},\tau ),\;\tau >0,$$

(15) $${\left.e^{\gamma^{*}}\frac{\partial \Theta^{*}(\zeta ,\tau )}{\partial \zeta }\right|}_{\zeta =1^{+}}={\left.K^{*}\frac{\partial \Theta^{*}(\zeta ,\tau )}{\partial \zeta }\right|}_{\zeta =1^{-}},\;\tau >0,$$

(16) $$\Theta^{*}(\zeta ,\tau )\to 0,\;\zeta \to \infty ,\;\tau >0,$$

(17) $$\Theta^{*}(\zeta ,0)=0,\;\zeta \geq 0.$$

## 3. Solution to the Problem

Applying the integral Laplace transform [37]:

(18) Θ¯*(ζ,p)≡L[Θ*(ζ,τ);p]=∫0∞Θ*(ζ,τ)e−pτdτ, Rep≥0,

into the problems (11)–(17), the following was obtained:

(19) $$\frac{d^{2}{\bar{\Theta }}^{*}(\zeta ,p)}{d\zeta^{2}}+\gamma^{*}\frac{d{\bar{\Theta }}^{*}(\zeta ,\tau )}{d\zeta }-pe^{-\gamma^{*}\zeta }{\bar{\Theta }}^{*}(\zeta ,p)=0,\;0<\zeta <1,$$

(20) $$\frac{d^{2}{\bar{\Theta }}^{*}(\zeta ,p)}{d\zeta^{2}}-\frac{p}{k^{*}}{\bar{\Theta }}^{*}(\zeta ,p)=0,\;\zeta >1,$$

(21) $${\left.\frac{d{\bar{\Theta }}^{*}(\zeta ,p)}{d\zeta }\right|}_{\zeta =0^{+}}=-\frac{1}{p},$$

(22) $${\bar{\Theta }}^{*}(1^{+},p)={\bar{\Theta }}^{*}(1^{-},p),$$

(23) $${\left.e^{\gamma^{*}}\frac{d{\bar{\Theta }}^{*}(\zeta ,p)}{d\zeta }\right|}_{\zeta =1^{+}}={\left.K^{*}\frac{d{\bar{\Theta }}^{*}(\zeta ,p)}{d\zeta }\right|}_{\zeta =1^{-}},$$

(24) $${\bar{\Theta }}^{*}(\zeta ,p)\to 0,\;\zeta \to \infty ,$$

The general solutions to the differential Equations (19) and (20) have the form:

(25) $${\bar{\Theta }}^{*}(\zeta ,p)=\xi \sqrt{p}[A_{1}(p)I_{1}(\xi \sqrt{p})+B_{1}(p)K_{1}(\xi \sqrt{p})],\;0\leq \zeta \leq 1,$$

(26) $${\bar{\Theta }}^{*}(\zeta ,p)=A_{2}(p)e^{-\varsigma \sqrt{p}}+B_{2}(p)e^{\varsigma \sqrt{p}},\;\zeta \geq 1,$$

where

(27) $$\xi =\frac{\alpha }{e^{\tilde{\alpha }\zeta }},\;\alpha =\frac{2}{\gamma^{*}},\;\tilde{\alpha }=\frac{1}{\alpha },\;\varsigma =\frac{\zeta -1}{\sqrt{k^{*}}},$$

$I_{n}(x)$, $K_{n}(x)$ here and further are modified Bessel functions of the *n*th order of the first and second kind, respectively [38]. Unknown functions $A_{l}(p)$ and $B_{l}(p)$, l=1,2 in the solutions (25), (26) were found from the boundary conditions (21)–(24) in the form:

(28) $$A_{1}(p)=\frac{\Delta_{A_{1}}(p)}{\alpha \;p^{2}\Delta (p)},\;B_{1}(p)=\frac{\Delta_{B_{1}}(p)}{\alpha \;p^{2}\Delta (p)},\;A_{2}(p)=\frac{e^{-\tilde{\alpha }}\Delta_{A_{2}}(p)}{p\sqrt{p}\Delta (p)},\;B_{2}(p)=0,$$

where

(29) $$\Delta (p)=\Delta_{A_{1}}(p{)I}_{0}(\alpha \sqrt{p})-\Delta_{B_{1}}(p{)K}_{0}(\alpha \sqrt{p}),$$

(30) $$\Delta_{A_{1}}(p)=K_{0}(\beta \sqrt{p})+\varepsilon \;e^{-\tilde{\alpha }}K_{1}(\beta \sqrt{p}),\;\Delta_{B_{1}}(p)=I_{0}(\beta \sqrt{p})-\varepsilon \;e^{-\tilde{\alpha }}I_{1}(\beta \sqrt{p}),$$

(31) $$\Delta_{A_{2}}(p)=I_{0}(\beta \sqrt{p})K_{1}(\beta \sqrt{p})+I_{1}(\beta \sqrt{p})K_{0}(\beta \sqrt{p})\equiv {\left(\beta \sqrt{p}\right)}^{-1},$$

(32) $$\beta =\frac{\alpha }{e^{\tilde{\alpha }}},\;\varepsilon =\frac{K^{*}}{\sqrt{k^{*}}}.$$

Considering Equations (28)–(32), solutions (25)–(27) took the form:

(33) $${\bar{\Theta }}^{*}(\zeta ,p)=e^{-\tilde{\alpha }\zeta }\;{\bar{\Theta }}_{0}^{*}(p){\bar{\Theta }}_{1}^{*}(\zeta ,p),0\leq \zeta \leq 1,\;{\bar{\Theta }}^{*}(\zeta ,p)=\tilde{\alpha }\;{\bar{\Theta }}_{0}^{*}(p){\bar{\Theta }}_{2}^{*}(\zeta ,p),\zeta \geq 1,$$

where

(34) $${\bar{\Theta }}_{0}^{*}(p)=\frac{1}{\sqrt{p}},\;{\bar{\Theta }}_{1}^{*}(\zeta ,p)=\frac{\Delta_{1}(\zeta ,p)}{p\Delta (p)},\;{\bar{\Theta }}_{2}^{*}(\zeta ,p)=\frac{e^{-\varsigma \sqrt{p}}}{p\sqrt{p}\Delta (p)},$$

(35) $$\Delta_{1}(\zeta ,p)=\Delta_{A_{1}}(p{)I}_{1}(\xi \sqrt{p})+\Delta_{B_{1}}(p{)K}_{1}(\xi \sqrt{p}).$$

Based on the convolution of two functions theorem [39], Formulas (33)–(35) yield:

(36) $$\Theta^{*}(\zeta ,\tau )=e^{-\tilde{\alpha }\zeta }\int_{0}^{\tau }\Theta_{0}^{*}(\tau -s)\Theta_{1}^{*}(\zeta ,s)ds,\;0\leq \zeta \leq 1,\;\tau \geq 0,$$

(37) $$\Theta^{*}(\zeta ,\tau )=\tilde{\alpha }\int_{0}^{\tau }\Theta_{0}^{*}(\tau -s)\Theta_{2}^{*}(\zeta ,s)ds,\;\zeta \geq 1,\;\tau \geq 0,$$

where

(38) $$\Theta_{0}^{*}(\tau )\equiv L^{-1}[{\bar{\Theta }}_{0}^{*}(p);\tau ]=\frac{1}{\sqrt{\pi \tau }},$$

(39) Θl∗(ζ,τ)≡L−1[Θ¯l∗(ζ,p); τ]=12πi∫ω−i ∞ω+i ∞Θ¯l∗(ζ,p)epτdp, l=1,2, ω≡Rep>0,i≡−1.

**Figure 2:**
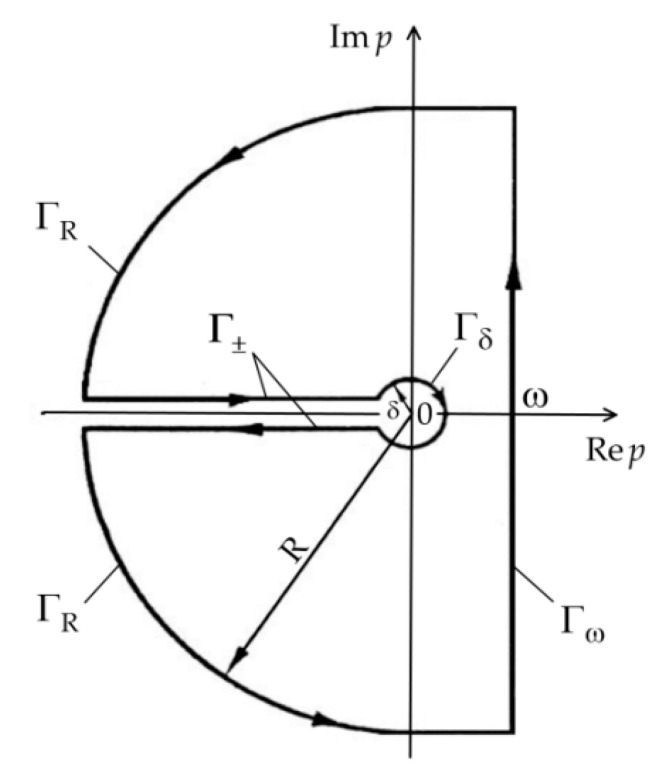
Integration contour.

Integration on the complex plane (Rep,  Im p) in Equation (39) was performed along the closed curve Γ (Figure 2). It consists of the segment $\Gamma_{\omega }$ of a straight line Re p=ω, the two arcs $\Gamma_{R}$ and $\Gamma_{\delta }$ centered at a point p=0 with radiuses R and δ, respectively, and a cut along the axis Re p<0 with boundaries $\Gamma_{\pm }$. Within the contour Γ the integral functions ${\bar{\Theta }}_{l}^{*}(\zeta ,p)$, l=1,2 (34) in the relation (39) are analytical and unambiguous, then by Cauchy’s theorem [40] we have:

(40) $$\frac{1}{2\pi i}\oint_{\Gamma }{\bar{\Theta }}_{l}^{*}(\zeta ,p)e^{p\tau }dp=0,\;l=1,2.$$

Since the functions ${\bar{\Theta }}_{l}^{*}(\zeta ,p)$, l=1,2 (34) satisfy the Jordan’s lemma conditions [37], integrals along the arc $\Gamma_{R}$ tend to be zero for R→∞ and from Equations (39) and (40), follows that:

(41) $$\Theta_{l}^{*}(\zeta ,\tau )=-\Theta_{l,+}^{*}(\zeta ,\tau )-\Theta_{l,-}^{*}(\zeta ,\tau )-\Theta_{l,\delta }^{*}(\zeta ,\tau ),\;\tau \geq 0,\;l=1,2,$$

where

(42) $$\Theta_{l,\pm }^{*}(\zeta ,\tau )=\frac{1}{2\pi i}\int_{\Gamma_{\pm }}{\bar{\Theta }}_{l}^{*}(\zeta ,p)e^{p\tau }dp,\;\Theta_{l,\delta }^{*}(\zeta ,\tau )=\frac{1}{2\pi i}\int_{\Gamma_{\delta }}{\bar{\Theta }}_{l}^{*}(\zeta ,p)e^{p\tau }dp,\;\tau \geq 0.$$

Proceeding to the polar system (r,ϕ) centered at a point p=0, parameter of the Laplace transform $p=re^{i\phi }$, r≥0, $\left|\phi \right|\leq \pi$. Then, on the boundaries $\Gamma_{\pm }$ we received $p=re^{\pm i\pi }=-r$, $\sqrt{p}=\pm i\sqrt{r}$ and the first integrals from Equation (42) can be written as:

(43) $$\Theta_{l,\pm }^{*}(\zeta ,\tau )=\pm \frac{1}{2\pi i}\int_{0}^{\infty }{\bar{\Theta }}_{l,\pm }^{*}(\zeta ,r)e^{-r\tau }dr,\;\tau \geq 0,\;l=1,2,$$

where ${\bar{\Theta }}_{l,\pm }^{*}(\zeta ,r)\equiv {\bar{\Theta }}_{l}^{*}(\zeta ,re^{\pm i\pi })$.

With consideration of the relations [38]:

(44) $$I_{0}(\pm ix)=J_{0}(x),\;K_{0}(\pm ix)=-0.5\pi [Y_{0}(x)\pm iJ_{0}\left(x\right)],$$

(45) $$I_{1}(\pm ix)=\pm iJ_{1}(ix),\;K_{1}(\pm ix)=-0.5\pi [J_{1}(x)\mp iY_{1}\left(x)\right],$$

where $J_{n}(x)$ and $Y_{n}(x)$ are Bessel functions of the *n*th order of the first and second kind, respectively, from formulae (29)–(35) it was found:

(46) $${\bar{\Theta }}_{1,\pm }^{*}(\zeta ,r)=\frac{\Delta_{1}^{\pm }(\zeta ,r)}{r\;\Delta^{\pm }(r)},\;0\leq \zeta \leq 1,\;{\bar{\Theta }}_{2,\pm }^{*}(\zeta ,r)=\frac{\Delta_{2}^{\pm }(\zeta ,r)}{r\sqrt{r}\;\Delta^{\pm }(r)},\;\zeta \geq 1,$$

where

(47) $$\Delta^{\pm }(r)=0.5\pi [\Delta_{R}(r)\mp i\varepsilon \;e^{-\tilde{\alpha }}\Delta_{I}(r)],$$

(48) $$\Delta_{1}^{\pm }(\zeta ,r)=0.5\pi [\varepsilon \;e^{-\tilde{\alpha }}\Delta_{1,R}(\zeta ,r)\pm i\Delta_{1,I}(\zeta ,r)],$$

(49) $$\Delta_{2}^{\pm }(\zeta ,r)=\sin(\varsigma \sqrt{r})\pm i\cos(\varsigma \sqrt{r}),$$

(50) $$\Delta_{R}(r)=Y_{0}(\alpha \sqrt{r})J_{0}(\beta \sqrt{r})-J_{0}(\alpha \sqrt{r})Y_{0}(\beta \sqrt{r}),$$

(51) $$\Delta_{I}(r)=Y_{0}(\alpha \sqrt{r})J_{1}(\beta \sqrt{r})-J_{0}(\alpha \sqrt{r})Y_{1}(\beta \sqrt{r}),$$

(52) $$\Delta_{1,R}(\zeta ,r)=Y_{1}(\beta \sqrt{r})J_{1}(\xi \sqrt{r})-J_{1}(\beta \sqrt{r})Y_{1}(\xi \sqrt{r}),$$

(53) $$\Delta_{1,I}(\zeta ,r)=Y_{0}(\beta \sqrt{r})J_{1}(\xi \sqrt{r})-J_{0}(\beta \sqrt{r})Y_{1}(\xi \sqrt{r}),$$

parameters α, $\tilde{\alpha }$, ξ, ς are determined by Equations (27), and β, ε by (32).

On the circular arc $\Gamma_{\delta }$ there is $p=\delta e^{i\phi }$, $\sqrt{p}=\sqrt{\delta }e^{0.5i\phi }$, $\left|\phi \right|\leq \pi$. Approaching the limit δ→0, the last integral (42) take the form:

(54) $$\Theta_{l,\delta }^{*}(\zeta ,\tau )=-\frac{1}{2\pi i}\underset{\delta \to 0}{\lim}\left(\int_{-\pi }^{\pi }{\bar{\Theta }}_{l,\delta }^{*}(\zeta ,\delta e^{i\phi })e^{\delta e^{i\phi \;}\tau }i\delta e^{i\phi }d\phi \right),\;\tau \geq 0,\;l=1,2,$$

where, with account of the solutions (34) we obtain:

(55) $${\bar{\Theta }}_{1,\delta }^{*}(\zeta ,\delta e^{i\phi })=\frac{\Delta_{1}(\zeta ,\delta e^{i\phi })}{\delta e^{i\phi }\Delta (\delta e^{i\phi })},0\leq \zeta \leq 1,\;{\bar{\Theta }}_{2,\delta }^{*}(\zeta ,\delta e^{i\phi })=\frac{e^{-\varsigma \sqrt{\delta }e^{0.5i\phi }}}{\delta \sqrt{\delta }e^{1.5i\phi }\Delta (\delta e^{i\phi })},\zeta \geq 1,$$

and functions $\Delta (\delta e^{i\phi })$ and $\Delta_{1}(\zeta ,\delta e^{i\phi })$ can be obtained from Equations (29)–(32) and (35).

Since for small values of the argument [38]:

(56) $$I_{0}(x)≅1,\;K_{0}(x)≅-\ln x,\;I_{1}(x)≅0.5x,\;K_{1}(x)≅x^{-1},$$

from formulae (29)–(32) and (35) the following was found:

(57) $$\Delta (\delta e^{i\phi })≅\frac{\gamma^{*}}{2}\left(1+\frac{\varepsilon }{\sqrt{\delta }e^{0.5i\phi }}\right),\Delta_{1}(\zeta ,\delta e^{i\phi })≅\frac{\varepsilon }{2}\left(e^{-0.5\gamma^{*}\zeta }-e^{-\gamma^{*}(1-0.5\zeta )}\right)+\frac{\gamma^{*}e^{0.5\gamma^{*}\zeta }}{2\sqrt{\delta }e^{0.5i\phi }},$$

Substituting the expressions (55)–(57) into the right side of the Equality (54) yields:

(58) $$\Theta_{1,\delta }^{*}(\zeta ,\tau )=-\frac{e^{0.5\gamma^{*}\zeta }}{\varepsilon },\;0\leq \zeta \leq 1,\;\Theta_{2,\delta }^{*}(\zeta ,\tau )=-\frac{2}{\varepsilon \gamma^{*}},\;\zeta \geq 1,\;\tau \geq 0.$$

Applying functions $\Theta_{l,\pm }^{*}(\zeta ,\tau )$ (43), (46)–(53), and $\Theta_{l,\delta }^{*}(\zeta ,\tau )$ (58) to the Equation (41), with consideration of the designations $\sqrt{r}=x$, $r=x^{2}$, it was obtained:

(59) $$\Theta_{1}^{*}(\zeta ,\tau )=\frac{e^{0.5\gamma^{*}\zeta }}{\varepsilon }+\frac{2}{\pi }\int_{0}^{\infty }\frac{\Delta_{1}(\zeta ,x)}{x\Delta (x)}e^{-x^{2}\tau }dx,\;0\leq \zeta \leq 1,\;\tau \geq 0,$$

(60) $$\Theta_{2}^{*}(\zeta ,\tau )=\frac{\alpha }{\varepsilon }-\frac{4}{\pi^{2}}\int_{0}^{\infty }\frac{\Delta_{2}(\zeta ,x)}{x^{2}\Delta (x)}e^{-x^{2}\tau }dx,\;\zeta \geq 1,\;\tau \geq 0,$$

where

(61) $$\Delta_{1}(\zeta ,x)=\Delta_{R}(x)\Delta_{1,I}(\zeta ,x)+\varepsilon^{2}e^{-\gamma^{*}}\Delta_{I}(x)\Delta_{1,R}(\zeta ,x),$$

(62) $$\Delta_{2}(\zeta ,x)=\Delta_{R}(x)\cos(\varsigma \;x)+\varepsilon \;e^{-0.5\gamma^{*}}\Delta_{I}(x)\sin(\varsigma \;x),$$

(63) $$\Delta (x)=\Delta_{R}^{2}(x)+\varepsilon^{2}e^{-\gamma^{*}}\Delta_{I}^{2}(x)],$$

(64) $$\Delta_{R}(x)=Y_{0}(\alpha x)J_{0}(\beta x)-J_{0}(\alpha x)Y_{0}(\beta x),$$

(65) $$\Delta_{I}(x)=Y_{0}(\alpha x)J_{1}(\beta x)-J_{0}(\alpha x)Y_{1}(\beta x),$$

(66) $$\Delta_{1,R}(\zeta ,x)=J_{1}(\beta x)Y_{1}(\xi x)-Y_{1}(\beta x)J_{1}(\xi x),$$

(67) $$\Delta_{1,I}(\zeta ,x)=J_{0}(\beta x)Y_{1}(\xi x)-Y_{0}(\beta x)J_{1}(\xi x).$$

Taking into account the functions $\Theta_{0}^{*}(\tau )$ (38) and $\Theta_{l}^{*}(\zeta ,\tau )$, l=1,2 (59), (60) in the relations (36) and (37), after integration, the sought dimensionless temperature rise was found in the form:

(68) $$\Theta^{*}(\zeta ,\tau )=2\sqrt{\frac{\tau }{\pi }}\left[\frac{1}{\varepsilon }+\frac{1}{\sqrt{\pi }}e^{-0.5\gamma^{*}\zeta }\int_{0}^{\infty }\frac{\Delta_{1}(\zeta ,x)}{x\Delta (x)}F(x\sqrt{\tau })dx\right],\;0<\zeta \leq 1,\;\tau \geq 0,$$

(69) $$\Theta^{*}(\zeta ,\tau )=2\sqrt{\frac{\tau }{\pi }}\left[\frac{1}{\varepsilon }-\frac{\gamma^{*}}{\pi \sqrt{\pi }}\int_{0}^{\infty }\frac{\Delta_{2}(\zeta ,x)}{x^{2}\Delta (x)}F(x\sqrt{\tau })dx\right],\;\zeta \geq 1,\;\tau \geq 0,$$

where

(70) $$F(x)=\frac{2e^{-x^{2}}}{\sqrt{\pi }x}\int_{0}^{x}e^{s^{2}}ds.$$

To calculate function F(x) (70), we used the following approximation formulae [41]:

(71) $$F(x)=\frac{2}{\sqrt{\pi }}\sum_{n=0}^{\infty }{\left(-1\right)}^{n}\frac{{\left(2x^{2}\right)}^{n}}{(2n+1)!!},\;0<x<3,\;F(x)=\frac{2}{\sqrt{\pi }}\sum_{n=0}^{N}\frac{(2n-1)!!}{{\left(2x^{2}\right)}^{n+1}},\;x\geq 3,$$

where (−1)!!=1, (2n+1)!!=1⋅3⋅5⋅...⋅(2n+1).

Besides the exact (in quadratures) solutions (68)–(71), the corresponding asymptotic solutions were also obtained for small and large values of the Fourier number (dimensionless time) τ.

*Small values of* τ (*large values of the parameter* p). Taking into account in Equations (29), (30), and (35) asymptotes of the modified Bessel functions for large values of the argument [38]:

(72) $$I_{n}(x)≅\frac{e^{x}}{\sqrt{2\pi x}},\;K_{n}(x)≅\sqrt{\frac{\pi }{2x}}\;e^{-x},\;n=0,\;1,\;....\;,$$

transforms (33) and (34) were obtained in the form:

(73) $${\bar{\Theta }}^{*}(\zeta ,p)≅e^{-0.25\gamma^{*}\zeta }\frac{e^{-(\alpha -\xi )\sqrt{p}}}{p\sqrt{p}},\;0\leq \zeta <1,$$

(74) $${\bar{\Theta }}^{*}(\zeta ,p)≅\frac{2e^{-0.25\gamma^{*}}}{\left(1+\varepsilon \;e^{-0.5\gamma^{*}}\right)}\frac{e^{-(\alpha -\beta +\varsigma )\sqrt{p}}}{p\sqrt{p}},\;\zeta \geq 1,$$

where, based on the definitions (27) and (32), we have:

(75) $$\alpha -\xi =\frac{2}{\gamma^{*}}(1-e^{-0.5\gamma^{*}\zeta })>0,\;\alpha -\beta +\varsigma =\frac{2}{\gamma^{*}}(1-e^{-0.5\gamma^{*}})+\frac{\zeta -1}{\sqrt{k^{*}}}>0.$$

Proceeding from the transforms (73) and (74) to the originals [39], asymptotes of dimensionless rise of temperature at the initial moments of the heating process were obtained in the form:

(76) $$\Theta^{*}(\zeta ,\tau )≅2\sqrt{\tau \;}e^{-0.25\gamma^{*}\zeta }\mathrm{ierfc}\left(\frac{\alpha -\xi }{2\sqrt{\tau }}\right),\;0\leq \zeta <1,\;0\leq \tau <<1,$$

(77) $$\Theta^{*}(\zeta ,\tau )≅\frac{4\sqrt{\tau \;}e^{-0.25\gamma^{*}}}{\left(1+\varepsilon \;e^{-0.5\gamma^{*}}\right)}\;\mathrm{ierfc}\left(\frac{\alpha -\beta +\varsigma }{2\sqrt{\tau }}\right),\;\zeta \geq 1,\;0\leq \tau <<1,$$

where, $\mathrm{ierfc}(x)=\pi^{-0.5}e^{-x^{2}}-x\mathrm{erfc}(x)$ erfc(x)=1−erf(x), erf(x)—Gauss error function [38].

*Large values of*τ (*small values of the parameter* p). For small values of the argument of modified Bessel functions with account of the expressions (56) and (57) Laplace transforms (33) and (34) were presented as:

(78) $${\bar{\Theta }}^{*}(\zeta ,p)≅\frac{\varepsilon \chi }{p(\sqrt{p}+\varepsilon )}+\frac{1}{p\sqrt{p}(\sqrt{p}+\varepsilon )},\;\;0\leq \zeta <1,\;\chi =\frac{1}{\gamma^{*}}(e^{-\gamma^{*}\zeta }-e^{-\gamma^{*}}),$$

(79) $${\bar{\Theta }}^{*}(\zeta ,p)≅\frac{e^{-\varsigma \sqrt{p}}}{p\sqrt{p}(\sqrt{p}+\varepsilon )},\;\zeta \geq 1,$$

where the coefficient ς is defined in Equation (27). Proceeding to the space of originals [39], the following asymptotes of dimensionless temperature rise for large values of the Fourier number τ:

(80) $$\Theta^{*}(\zeta ,\tau )≅\frac{2}{\varepsilon }\sqrt{\frac{\tau }{\pi }}+\left(\chi -\frac{1}{\varepsilon^{2}}\right)\left[1-e^{\varepsilon^{2}\tau }\mathrm{erfc}(\varepsilon \sqrt{\tau })\right],\;0\leq \zeta <1,\;\tau >>1,$$

(81) $$\Theta^{*}(\zeta ,\tau )≅\frac{2}{\varepsilon }\sqrt{\frac{\tau }{\pi }}\;e^{-\frac{\varsigma^{2}}{4\tau }}-\frac{1}{\varepsilon }\left(\varsigma +\frac{1}{\varepsilon }\right)\mathrm{erfc}\left(\frac{\varsigma }{2\sqrt{\tau }}\right)+\frac{1}{\varepsilon^{2}}e^{\varepsilon \varsigma +\varepsilon^{2}\tau }\mathrm{erfc}\left(\frac{\varsigma }{2\sqrt{\tau }}+\varepsilon \sqrt{\tau }\right),\;\;\zeta \geq 1,\;\tau >>1.$$

On the interface ζ=1, coefficients are χ=ς=0 and from the Equations (80) and (81) it was obtained:

(82) $$\Theta^{*}(1^{+},\tau )=\Theta^{*}(1^{-},\tau )≅\frac{2}{\varepsilon }\sqrt{\frac{\tau }{\pi }}-\frac{1}{\varepsilon^{2}}\left[1-e^{\varepsilon^{2}\tau }\mathrm{erfc}(\varepsilon \sqrt{\tau })\right].$$

## 4. Verification of the Solution

We will prove that the derived solution (68)–(70) satisfies the boundary conditions (13)–(16) and the initial condition (17). After differentiating the solution (68) with respect to the spatial variable ζ, it was obtained:

(83) $$\frac{\partial \Theta^{*}(\zeta ,\tau )}{\partial \zeta }=2\sqrt{\frac{\tau }{\pi }}\;\left[\frac{1}{\sqrt{\pi }}e^{-0.5\gamma^{*}\zeta }\int_{0}^{\infty }\frac{\left[{\Delta_{1}}^{\prime }(\zeta ,x)-0.5\gamma^{*}\Delta_{1}(\zeta ,x)\right]}{x\Delta (x)}F(x\sqrt{\tau })dx\right],\;0\leq \zeta \leq 1,\;\;\tau \geq 0,$$

where derivative of the function $\Delta_{1}(\zeta ,x)$ (61) has the form:

(84) $${\Delta_{1}}^{\prime }(\zeta ,x)=\Delta_{R}(x){\Delta_{1,I}}^{\prime }(\zeta ,x)+\varepsilon^{2}e^{-\gamma^{*}}\Delta_{I}(x){\Delta_{1,R}}^{\prime }(\zeta ,x).$$

Considering derivatives [38]:

(85) $$J_{1}^{\prime }(x)=J_{0}(x)-x^{-1}J_{1}(x),\;Y_{1}^{\prime }(x)=Y_{0}(x)-x^{-1}Y_{1}(x),$$

and Equations (64)–(67) for ζ=0 it was found:

(86) $${\Delta_{1,R}}^{\prime }(0,x)=0.5\gamma^{*}\Delta_{1,R}(0,x)-x\Delta_{I}(x),\;{\Delta_{1,I}}^{\prime }(0,x)=0.5\gamma^{*}\Delta_{1,I}(0,x)-x\Delta_{R}(x),$$

(87) $$\Delta_{1,R}(0,x)=Y_{1}(\alpha \;x)J_{1}(\beta \;x)-J_{1}(\alpha \;x)Y_{1}(\beta x),\;\Delta_{1,I}(0,x)=Y_{1}(\alpha \;x)J_{0}(\beta x)-J_{1}(\alpha \;x)Y_{0}(\beta x),$$

Substituting the Equations (86) and (87) to the expression (84), it was obtained:

(88) $${\Delta_{1}}^{\prime }(0,x)=0.5\gamma^{*}\Delta_{1}(0,x)-x\Delta (x),$$

where functions $\Delta_{1}(0,x)$ and Δ(x) were determined from Equations (61) and (63), respectively. Next, from the Equation (83), with consideration of the derivative (88), the following was found:

(89) $${\left.\frac{\partial \Theta^{*}(\zeta ,\tau )}{\partial \zeta }\right|}_{\zeta =0^{+}}=-\frac{2}{\sqrt{\pi }}\sqrt{\frac{\tau }{\pi }}\;\int_{0}^{\infty }F(x\sqrt{\tau })dx,\;\tau \geq 0,$$

where function F(x) has form (69), (70). Taking into account the value of the integral [41]:

(90) $$\int_{0}^{\infty }F(x)dx=\frac{\pi }{2},$$

from the Equation (88) it follows that boundary condition (13) is met.

Comparing the forms of solutions (68) and (69) it can be concluded that the boundary condition (14) is satisfied, when:

(91) $$e^{-0.5\gamma^{*}}\Delta_{1}(1,x)=-\frac{\gamma^{*}\Delta_{2}(1,x)}{\pi x}.$$

Taking into consideration that for ζ=1 yields ξ=β and ς=0, from Equations (61)–(67) it was obtained:

(92) $$\Delta_{1,R}(1,x)=0,\;\Delta_{1}(1,x)=\Delta_{R}(x)\Delta_{1,I}(1,x),\;\Delta_{2}(1,x)=\Delta_{R}(x).$$

Then, using relations [38]:

(93) $$\Delta_{1,I}(1,x)=J_{0}(\beta x)Y_{1}(\beta \;x)-Y_{0}(\beta x)J_{1}(\beta \;x)=-\frac{2}{\pi \beta x},$$

definition of coefficient β (27), (32) and Equation (92), the Equality (91) takes the form:

(94) $$\frac{\gamma^{*}}{\pi x}\Delta_{R}(x)\equiv \frac{\gamma^{*}}{\pi x}\Delta_{R}(x),$$

which confirm the fulfillment of the boundary condition (14).

For ζ=1 from Equations (83) and (84), it was found:

(95) $${\left.\frac{\partial \Theta^{*}(\zeta ,\tau )}{\partial \zeta }\right|}_{\zeta =1^{+}}=2\sqrt{\frac{\tau }{\pi }}\;\left[\frac{1}{\sqrt{\pi }}e^{-0.5\gamma^{*}}\int_{0}^{\infty }\frac{\left[{\Delta_{1}}^{\prime }(1,x)-0.5\gamma^{*}\Delta_{1}(1,x)\right]}{x\Delta (x)}F(x\sqrt{\tau })dx\right],\;\tau \geq 0,$$

(96) $${\Delta_{1}}^{\prime }(1,x)=\Delta_{R}(x){\Delta_{1,I}}^{\prime }(1,x)+\varepsilon^{2}e^{-\gamma^{*}}\Delta_{I}(x){\Delta_{1,R}}^{\prime }(1,x),$$

where the corresponding values of the derivatives of the function $\Delta_{1,R}(\zeta ,x)$ (66) and $\Delta_{1,I}(\zeta ,x)$ (67) with respect to the variable ζ have the form:

(97) $${\Delta_{1,R}}^{\prime }(1,x)=-\frac{\gamma^{*}}{\pi },\;{\Delta_{1,I}}^{\prime }(1,x)=-\frac{\gamma^{*}}{\pi \beta x},$$

Substituting the above Equation (97) to the right side of Equality (96), and next, considering in this manner the obtained derivative ${\Delta_{1}}^{\prime }(1,x)$ and functions $\Delta_{1}(1,x)$ (92), (93) in the integrand (95), it was received:

(98) $$e^{\gamma^{*}}{\left.\frac{\partial \Theta^{*}(\zeta ,\tau )}{\partial \zeta }\right|}_{\zeta =1^{+}}=-\frac{2}{\pi^{2}}\varepsilon^{2}\gamma^{*}e^{-0.5\gamma^{*}}\sqrt{\tau }\int_{0}^{\infty }\frac{\Delta_{I}(x)}{x\Delta (x)}F(x\sqrt{\tau })dx,\;\tau \geq 0.$$

On the other hand, differentiating the solution (69) with respect to the variable ζ yields:

(99) $${\left.K^{*}\frac{\partial \Theta^{*}(\zeta ,\tau )}{\partial \zeta }\right|}_{\zeta =1^{-}}=-\frac{2}{\pi^{2}}\gamma^{*}K^{*}\sqrt{\tau }\int_{0}^{\infty }\frac{{\Delta_{2}}^{\prime }(1,x)}{x^{2}\Delta (x)}F(x\sqrt{\tau })dx,\;\tau \geq 0,$$

where value of the derivative function $\Delta_{2}(\zeta ,x)$ (61) for ζ=1 is equal to:

(100) $${\Delta_{2}}^{\prime }(1,x)=\frac{\varepsilon \;e^{-0.5\gamma^{*}}}{\sqrt{k^{*}}}x\Delta_{I}(x).$$

Taking into consideration of derivative (100) and definition (32) of thermal activity ε in the right side of the equality (99), it was established that it is the same as the right side of Equation (98). In this way, it was proved that the obtained solutions (68)–(70) meet the boundary condition (15). Fulfillment of the condition (16) of temperature rise disappearance (69) for ζ→∞ is guaranteed by omitting the function $B_{2}(p)$ (28) in Equation (26). Additionally, it was checked during the numerical calculations. Obviously, the solutions (68)–(70) satisfy also the initial condition (17).

## 5. Heating the Coating Surface by a Heat Flux with Linearly Decreasing Intensity in Time

Presented above exact solutions (68)–(70) were developed for constant intensity of heat flux $q_{0}$ over time. In this chapter, the heating process of the FGC surface deposited on the homogeneous substrate, with time-dependent heat flux intensity:

(101) $$q(t)=q_{0}q^{*}(t),\;q^{*}(t)=1-t\;t_{s}^{-1},\;0\leq t\leq t_{s},$$

where $t_{s}$—the final moment of heating process. It should be noted that the evolution of the heat flux intensity in the form (101) is characteristic for thermal problems of friction formulated for braking process with constant deceleration [42]. Dimensionless temperature rise ${\hat{\Theta }}^{*}(\zeta ,\tau )$, corresponding to the heat flux intensity (101) can be found based on Duhamel’s theorem [43]:

(102) $${\hat{\Theta }}^{*}(\zeta ,\tau )=\frac{\partial }{\partial \tau }\int_{0}^{\tau }q^{*}(\tau -s)\Theta^{*}(\zeta ,s)ds,\;\zeta \geq 0,\;0\leq \tau \leq \tau_{s},$$

where $\Theta^{*}(\zeta ,\tau )$ is dimensionless temperature rise (68)–(70), and function $q^{*}(\tau )$ has the form:

(103) $$q^{*}(\tau )=1-\tau \;\tau_{s}^{-1},\;0\leq \tau \leq \tau_{s},\;\tau_{s}=k_{1}t_{s}d^{-2}.$$

Substituting the solution (68) and function $q^{*}(\tau )$ (103) to the integrand in the expression (102), it was found:

(104) $${\hat{\Theta }}^{*}(\zeta ,\tau )=\frac{\partial }{\partial \tau }\left[\frac{2}{\sqrt{\pi }\varepsilon }Q_{1}(\tau )+\frac{2}{\pi }e^{-0.5\gamma^{*}\zeta }\int_{0}^{\infty }\frac{\Delta_{1}(\zeta ,x)}{x\Delta (x)}Q_{2}(\tau ,x)dx\right],0\leq \zeta \leq 1,\;\;0\leq \tau \leq \tau_{s},$$

where

(105) $$Q_{1}(\tau )=\int_{0}^{\tau }\left(1-\frac{\tau -s}{\tau_{s}}\right)\sqrt{s}ds=\frac{2}{3}\tau \sqrt{\tau }\left(1-\frac{2\tau }{5\tau_{s}}\right),$$

(106) $$Q_{2}(\tau ,x)=\int_{0}^{\tau }\left(1-\frac{\tau -s}{\tau_{s}}\right)\sqrt{s}F(x\sqrt{s})ds=\left(1-\frac{\tau }{\tau_{s}}\right)Q_{21}(\tau ,x)+\frac{1}{\tau_{s}}Q_{22}(\tau ,x),$$

(107) $$Q_{21}(\tau ,x)=\int_{0}^{\tau }\sqrt{s}F(x\sqrt{s})ds,\;Q_{22}(\tau ,x)=\int_{0}^{\tau }s\sqrt{s}F(x\sqrt{s})ds.$$

Taking into consideration the function F(x) (70), integrals (107) were written in the form:

(108) $$Q_{21}(\tau ,x)=\frac{2}{x^{2}}\sqrt{\frac{\tau }{\pi }}\left[1-\frac{\sqrt{\pi }}{2}F(x\sqrt{\tau })\right],$$

(109) $$Q_{22}(\tau ,x)=\frac{2}{x^{4}}\sqrt{\frac{\tau }{\pi }}\left[1+\frac{1}{3}x^{2}\tau -\frac{\sqrt{\pi }}{2}(1+x^{2}\tau )F(x\sqrt{\tau })\right].$$

Applying functions $Q_{21}(\tau ,x)$ (108) and $Q_{22}(\tau ,x)$ (109) into Equation (106), it was received:

(110) $$Q_{2}(\tau ,x)=\frac{2}{x^{2}}\sqrt{\frac{\tau }{\pi }}\left[1-\frac{2\tau }{3\tau_{s}}+\frac{1}{x^{2}\tau_{s}}-\frac{\sqrt{\pi }}{2}\left(1+\frac{1}{x^{2}\tau_{s}}\right)F(x\sqrt{\tau })\right].$$

Next, differentiating the functions $Q_{1}(\tau )$ (105) and $Q_{2}(\tau ,x)$ (110), it was found:

(111) $$P_{1}(\tau )\equiv \frac{dQ_{1}(\tau )}{d\tau }=\sqrt{\tau }\;P_{1}^{*}(\tau ),\;P_{1}^{*}(\tau )=1-\frac{2\tau }{3\tau_{s}},$$

(112) $$P_{2}(\tau ,x)\equiv \frac{\partial Q_{2}(\tau ,x)}{\partial \tau }=\sqrt{\tau }\;P_{2}^{*}(\tau ,x),\;P_{2}^{*}(\tau ,x)=\left(1+\frac{1}{x^{2}\tau_{s}}\right)F(x\sqrt{\tau })-\frac{2}{\sqrt{\pi }x^{2}\tau_{s}}.$$

From the Duhamel’s formula (104) with account of derivatives (111) and (112), it was obtained:

(113) $${\hat{\Theta }}^{*}(\zeta ,\tau )=2\sqrt{\frac{\tau }{\pi }}\left[\frac{1}{\varepsilon }P_{1}^{*}(\tau )+\frac{1}{\sqrt{\pi }}e^{-0.5\gamma^{*}\zeta }\int_{0}^{\infty }\frac{\Delta_{1}(\zeta ,x)}{x\Delta (x)}P_{2}^{*}(\tau ,x)dx\right],\;0\leq \zeta \leq 1,0\leq \tau \leq \tau_{s}.$$

Noting that solution (113) is structurally close (with accuracy to the functions $P_{1}^{*}(\tau )$ (111) i $P_{2}^{*}(\tau ,x)$ (112)) to the solution (68), based on the expression (69) the following can be written:

(114) $${\hat{\Theta }}^{*}(\zeta ,\tau )=2\sqrt{\frac{\tau }{\pi }}\left[\frac{1}{\varepsilon }P_{1}^{*}(\tau )-\frac{\gamma^{*}}{\pi \sqrt{\pi }}\int_{0}^{\infty }\frac{\Delta_{2}(\zeta ,x)}{x^{2}\Delta (x)}P_{2}^{*}(\tau ,x)dx\right],\;\zeta \geq 1,\;0\leq \tau \leq \tau_{s}.$$

It should be noted that for $\tau_{s}\to \infty$ from the Equations (111) and (112) follows that $P_{1}^{*}(\tau )=1$,$P_{2}^{*}(\tau ,x)=F(x\sqrt{\tau })$ and solutions (113), (114) become the same, as well as the previously obtained solutions (68), (69) at constant heat flux intensity.

## 6. Numerical Analysis

Calculations were performed for a coating made of two-component FGM, applied on the homogeneous substrate. On the outer surface of FGC is pure zirconium dioxide ZrO_2_ that smoothly transforms into a titanium alloy Ti-6Al-4V in the structure of the coating material. The element substrate is made of cast iron ChNMKh. Essential for calculations, properties of these materials at the ambient temperature $T_{0}=20{\;}^{\circ }C$ are included in Table 1.

Specific heat capacity and density of functionally graded coating material was determined according to the mixture law:

(115) $$c_{1}=c_{1,1}v+(1-v)c_{1,2},\;\rho_{1}=\rho_{1,1}v+(1-v)\rho_{1,2},$$

where 0≤v≤1 is the volume fracture of the FGM components. For the same proportion of both components (v=0.5) it was established that $c_{1}=495.55\;J\;{\mathrm{kg}}^{-1}K^{-1}$, $\rho_{1}=\;5266.98\mathrm{kg}\;m^{-3}$. Moreover from the Equation (9) the dimensionless values of thermal conductivity $K^{*}=26.89$ and diffusivity $k^{*}=22.23$ were found, and the thermal activity ε=5.7 from the Equation (32). Gradient parameter of considered FGM was found based on the following relation [45]:

(116) $$\gamma^{*}=\ln(\;K_{1,2}\;K_{1,1}^{\;-1})=1.26.$$

Main objects of the numerical analysis are dimensionless temperature rises $\Theta^{*}(\zeta ,\tau )$ (68), (69) and ${\hat{\Theta }}^{*}(\zeta ,\tau )$ (113), (114) initiated by heating the coating surface by heat fluxes with constant and linearly decreasing intensities over time. Numerical integration in the Equations (68), (69) and (113), (114) were performed utilizing the QAGI procedure from the package QUADPACK [46].

**Figure 3:**
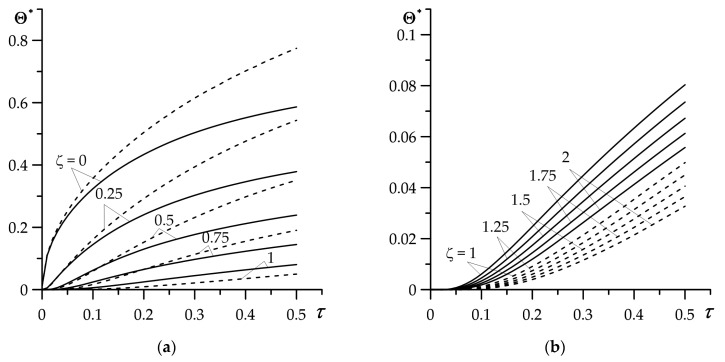
Evolutions of dimensionless temperature rise $\Theta^{*}(\zeta ,\tau )$ for selected values of dimensionless spatial variable ζ in: (**a**) coating, (**b**) substrate. Solid lines—FGC, dashed lines—homogeneous ZrO_2_ coating.

**Figure 4:**
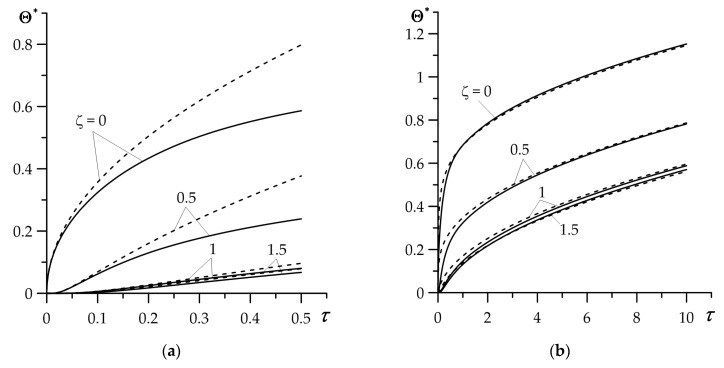
Comparison of time profiles of dimensionless temperature rise $\Theta^{*}(\zeta ,\tau )$ in the FGC, obtained using the exact (68), (69) (solid lines) and asymptotic (dashed lines) solutions: (**a**) for small (76), (77), (**b**) for large (80), (81) Fourier number τ, for selected values of dimensionless spatial variable ζ.

Variations of dimensionless temperature rise $\Theta^{*}(\zeta ,\tau )$ (68), (69) over time of heating (Fourier number τ) in the coating and substrate is presented in Figure 3 and Figure 4. Deposition of a FGC on a substrate causes a drop of temperature compared to a homogeneous coating made entirely of zirconium dioxide (Figure 3a). It is valuable that this effect becomes more noticeable with the passage of heating time, and thus with increasing the temperature of the protective layer. So, the Ti-6Al-4V titanium alloy, having a thermal conductivity three times higher than zirconium dioxide ZrO_2_, fulfills well the assumed *a priori* role of dissipating heat from the heated surface of the FGC and thus lowering its temperature. It should be noted that the described effect of lowering the temperature as a result of using FGM is maintained inside the layer (0≤ζ<1). However, starting from the interface and further into the substrate (ζ≥1), we observe the opposite behavior of the temperature evolution—the temperature of the substrate with the FGC is higher than when using a homogeneous coating (Figure 3b). This is due to the significantly higher thermal conductivity of cast iron compared to the titanium alloy (Table 1). The temperature of the substrate is much (more than an order of magnitude) lower than the temperature of the coating.

Results demonstrated in Figure 3 were obtained based on exact solutions for coating (68) and substrate (69), whereas Figure 4 illustrates the outcomes established by means of the asymptotic solutions for small (76), (77) and large (80), (81) values of dimensionless time (Fourier number) τ. Calculations were performed only for the FGC.

Satisfactory agreement of exact and asymptotic (for small τ) solutions takes place in the range 0≤τ≤0.1 (Figure 4a). On the other hand, the good agreement of the results obtained with the use of exact and asymptotic (for large τ) solutions occurs at τ≥1 (Figure 4b). The advantage of asymptotic solutions is their analytical form, which eliminates the need for laborious procedures of numerical integration. Thus, the developed asymptotic solutions can be used for expressing the estimation of the temperature mode of the considered coating–substrate system.

**Figure 5:**
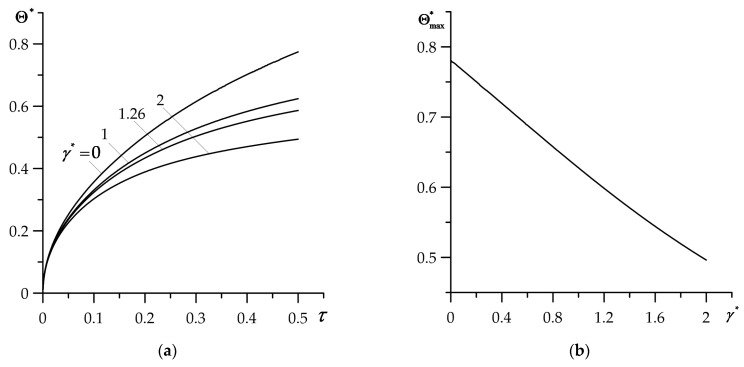
Effect of FGC gradient $\gamma^{*}$ on dimensionless temperature rise: (**a**) evolution $\Theta^{*}(0,\tau )$ (68), (69) for selected values of $\gamma^{*}$, (**b**) dependency $\Theta_{\max}^{*}\equiv \Theta^{*}(0,\;0.5)$ on $\gamma^{*}$.

As shown in Figure 3 and Figure 4, the outcomes were obtained for FGM gradient parameter $\gamma^{*}=1.26$ determined based on the Equation (116). On the other hand, $\gamma^{*}$ can be considered as independent input parameter, responsible for the speed of “transition” of the thermal conductivity coefficient $K_{1}$ of coating material, from the value $K_{1,1}$ of the first component to the value $K_{1,2}$ of the second component, according to the formula (1). Influence of the gradient $\gamma^{*}$ on the temperature of heated coating surface is presented in Figure 5, where results obtained for $\gamma^{*}=0$ correspond to the coating made entirely from the zirconium dioxide. Increase in FGM gradient causes a drop in temperature, which becomes more noticeable as the heating process progresses (Figure 5a). At the final moment τ=0.5, the decrease in the temperature of the heated FGC surface is almost linear (Figure 5b).

**Figure 6:**
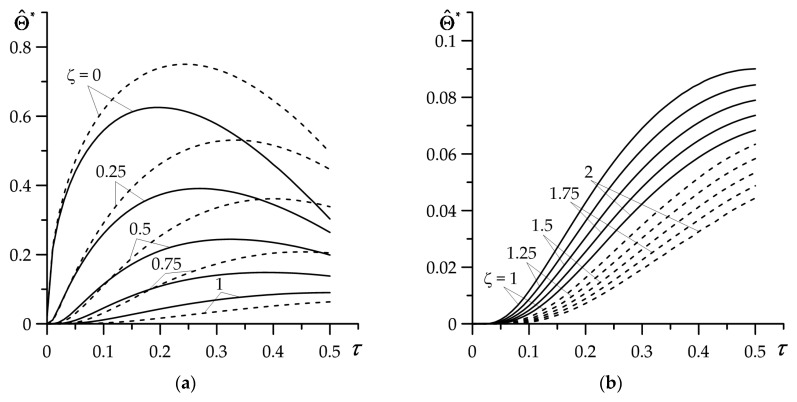
Evolution of dimensionless temperature rise ${\hat{\Theta }}^{*}(\zeta ,\tau )$ (113), (114) for selected dimensionless values of spatial variable ζ in: (**a**) coating, (**b**) substrate. Solid lines—FGC, dashed lines—homogeneous coating made of ZrO_2_.

Investigation of the impact of linearly decreasing time profile of the heat flux intensity (103) was carried out based on solutions (113) and (114) for $\tau_{s}=0.5$, $\gamma^{*}=1.26$ (Figure 6). For a constant intensity of the heat flux, the temperature of the coating and substrate increases monotonically with the heating time (Figure 3). However, in the considered case of a linear heat flux intensity profile, the temperature of the coating reaches its maximum value ${\hat{\Theta }}_{\max}^{*}$ at the moment of time $0<\tau_{\max}<\tau_{s}$, and then decreases (Figure 6a). The maximum temperature value ${\hat{\Theta }}_{\max}^{*}$ is achieved the earliest on the FGC outer surface ζ=0. The time to reach ${\hat{\Theta }}_{\max}^{*}$ increases with the distance from heated surface.

Starting from the interface ζ=1 and further into the substrate ζ>1, the temperature increases throughout the heating process, reaching the highest value at the moment $\tau_{\max}=\tau_{s}$ (Figure 6b). Both at a constant and at the time-dependent intensity of the heat flux, consideration of the FGC gradient causes a drop in the temperature value in relation to the temperature of the coating made of a homogeneous material. In the substrate, the situation is opposite—at fixed value of ζ, the temperature is lower in case of a homogeneous coating material. Regardless of the coating material (FGM or homogeneous), the substrate temperature is an order lower than in the coating.

**Figure 7:**
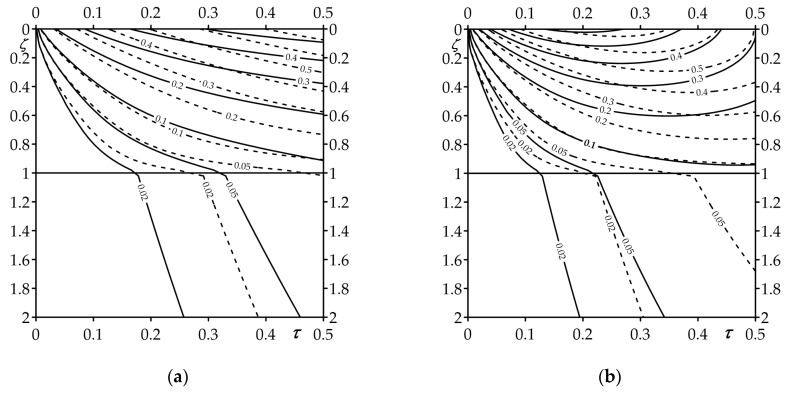
Dimensionless isolines of the temperature rises: (**a**) $\Theta^{*}(\zeta ,\tau )$ (68), (69), (**b**) ${\hat{\Theta }}^{*}(\zeta ,\tau )$(113), (114). Solid lines—FGC, dashed lines—homogeneous coating made of ZrO_2_.

Spatial-temporal distributions of temperature are presented in Figure 7, for constant and time-dependent heat flux intensities. Visible differences between the shape of the corresponding isotherms occur only in the more heated element of the system—the coating that absorbs the main part of the thermal load. In the case of a constant intensity of the heat flux, the isotherms with a fixed temperature level go deeper inside the FGC during the whole heating process. On the other hand, during heating by a heat flux with linearly decreasing intensity over time, the isotherms in the coating reach the maximum distance from the heated surface before the end of the process.

## 7. Conclusions

As a result of the performed analysis, it was found that:

1. Deposition of functionally graded coating on the homogeneous substrate allow to effectively lower the temperature on the heated surface;
2. FGC is the main adsorbent of frictional heat generated. As a result, values of temperature achieved in the substrate are much lower than that obtained in the coating temperature level;
3. The temporal profile of the heat flux intensity has a noticeable impact on the spatial-temporal distribution of isotherms only in the coating;
4. Gradient parameter of the FGC has a crucial influence on the maximum temperature for the selected coating–substrate system;
5. Obtained asymptotic solutions are useful for the express estimation of the temperature of the FGC-substrate system at small and large values of the Fourier number;
6. The proposed mathematical model can be utilized as an effective tool for simulating the temperature mode of homogeneous bodies with functionally graded coating.

In summary, it should be noted that the developed methodology for obtaining accurate and asymptotic solutions can be successfully applied also to the FGM class, not only with increasing but also decreasing thermal conductivity along the thickness, as well as in the problems of thermal conduction, considering the generation of heat due to friction on the contact surface of the protective layer with the counterbody. For example, such a pair may be formed by a brake disc coated with FGC in combination with a pad.

## Figures and Tables

**Table 1:** Materials properties [34,44].

Material	Thermal Conductivity ${\mathrm{Wm}}^{-1}K^{-1}$	Specific Heat Capacity $J\;{\mathrm{kg}}^{-1}K^{-1}$	Density $\;\mathrm{kg}\;m^{-3}$
ZrO_2_	$K_{1,1}=1.94$	$c_{1,1}=\;452.83$	$\rho_{1,1}=\;6102.16$
Ti-6Al-4V	$K_{1,2}=6.87$	$c_{1,2}=\;538.08$	$\rho_{1,2}=\;4431.79$
ChNMKh	$K_{2}=52.17$	$c_{2}=\;444.6$	$\rho_{2}=\;7100$

## Data Availability

No new data were created or analyzed in this study. Data sharing is not applicable to this article.

## Nomenclature

c	Specific heat capacity ($J\;{\mathrm{kg}}^{-1}K^{-1}$)
d	Thickness of FGC (m)
$I_{n}(\cdot )$	Modified Bessel functions of the *n*th order of the first kind
$J_{n}(\cdot )$	Bessel functions of the *n*th order of the first kind
$K_{n}(\cdot )$	Modified Bessel functions of the *n*th order of the second kind
k	Thermal diffusivity ($m^{2}s^{-1}$)
K	Thermal conductivity ($W\;m^{-1}K^{-1}$)
q	Intensity of heat flux ($W\;m^{-2}$)
$q_{0}$	Nominal value of the heat flux intensity ($W\;m^{-2}$)
t	Time (s)
$t_{s}$	Final moment of the heating process (s)
T	Temperature (°C)
$T_{0}$	Initial temperature (°C)
v	Volume fraction of the material phases (dimensionless)
$Y_{n}(\cdot )$	Bessel functions of the *n*th order of the second kind
z	Spatial coordinate in axial direction (m)
γ	Parameter of FGM gradient ($m^{-1}$)
$\gamma^{*}$	Dimensionless parameter of FGM gradient
Λ	Temperature rise scaling factor (°C)
ε	Dimensionless coefficient of thermal activity of friction couple
Θ	Temperature rise (°C)
$\Theta^{*}$	Dimensionless temperature rise
ρ	Density ($\mathrm{kg}\;m^{-3}$)
τ	Dimensionless time
$\tau_{s}$	Dimensionless final time of heating
ζ	Dimensionless spatial coordinate in axial direction

## References

[1] Alinia Y., Beheshti A., Guler M.A., El-Borgi S., Polycarpou A.A. (2016). Sliding contact analysis of functionally graded coating/substrate system. Mech. Mater..

[2] Kulchytsky-Zhyhailo R., Bajkowski A. (2012). Analytical and numerical methods of solution of three-dimensional problem of elasticity for functionally graded coated half-space. Int. J. Mech. Sci..

[3] Bishnoi D. (2023). Pressure exertion and heat dissipation analysis on uncoated and ceramic (Al_2_O_3_, TiO_2_ and ZrO_2_) coated braking pads. Mater. Today Proc..

[4] Yevtushenko A., Kuciej M., Och E. (2017). Influence of thermal sensitivity of the materials on temperature and thermal stresses of the brake disc with thermal barrier coating. Int. Commun. Heat Mass Transf..

[5] Varecha D., Bronček J., Kohar R., Nový F., Vicen M., Radek N. (2021). Research of friction materials applicable to the multi-disc brake concept. J. Mater. Res. Technol..

[6] Strojny-Nędza A., Pietrzak K., Gili F., Chmielewski M. (2020). FGM based on copper–alumina composites for brake disc applications. Arch. Civ. Mech. Eng..

[7] Govindaraju M., Megalingam A., Murugasan J., Vignesh R.V., Kota P.K., Ram A.S., Lakshana P., Kumar V.N. (2020). Investigations on the tribological behavior of functionally gradient iron-based brake pad material. Proc. Inst. Mech. Eng. Part C J. Mech. Eng. Sci..

[8] Babu K.V., Marichamy S., Ganesan P., Madan D., Uthayakumar M., Rajan T.P. (2020). Processing of functionally graded aluminum composite brake disc and machining parameters optimization. Mater. Today Proc..

[9] Pakseresht A.H., Rahimipour M.R., Alizadeh M., Hadavi S.M.M., Shahbazkhan A., Zuzuarregui A., Morant-Minana M.C. (2016). Concept of advanced thermal barrier functional coatings in high temperature engineering components. Research Perspectives on Functional Micro- and Nanoscale Coatings.

[10] Buyukkaya E. (2008). Thermal analysis of functionally graded coating AlSi alloy and steel pistons. Surf. Coat. Technol..

[11] Mondal K., Nuñez III L., Downey C.M., Van Rooyen I.J. (2021). Recent advances in the thermal barrier coatings for extreme environments. Mater. Sci. Energy Technol..

[12] Topczewska K. (2018). Analytical model for investigation of the effect of friction power on the thermal stresses in friction elements of brakes. J. Theor. Appl. Mech..

[13] Zhao J., Li Y., Ai X. (2008). Analysis of transient thermal stress in sandwich plate with functionally graded coatings. Thin Solid Film..

[14] Sathish M., Radhika N., Saleh B.A. (2021). critical review on functionally graded coatings: Methods, properties, and challenges. Compos. Part B Eng..

[15] Pasupuleti K.T., Dsouza S., Thejaraju R., Venkataraman S., Ramaswamy P., Murty S.N. (2018). Performance and steady state heat transfer analysis of functionally graded thermal barrier coatings systems. Mater. Today Proc..

[16] Suresh S. (2001). Graded materials for resistance to contact deformation and damage. Science.

[17] Khor K.A., Gu Y.W. (2000). Thermal properties of plasma-sprayed functionally graded thermal barrier coatings. Thin Solid Film..

[18] Balci M.N., Dag S., Yildirim B. (2017). Subsurface stresses in graded coatings subjected to frictional contact with heat generation. J. Therm. Stress..

[19] Balci M.N., Dag S. (2019). Solution of the dynamic frictional contact problem between a functionally graded coating and a moving cylindrical punch. Int. J. Solids Struct..

[20] Fu P., Zhao J., Zhang X., Kang G., Wang P., Kan Q. (2022). Thermo-mechanically coupled sliding contact shakedown analysis of functionally graded coating-substrate structures. Int. J. Mech. Sci..

[21] Jin Z.H. (2002). An asymptotic solution of temperature field in a strip a functionally graded material. Int. Commun. Heat Mass Transf..

[22] Jobin K.J., Abhilash M.N., Murthy H. (2017). A simplified analysis of 2D sliding frictional contact between rigid indenters and FGM coated substrates. Tribol. Int..

[23] Burlayenko V.N., Altenbach H., Sadowski T., Dimitrova S.D., Bhaskar A. (2017). Modelling functionally graded materials in heat transfer and thermal stress analysis by means of graded finite elements. Appl. Math. Model..

[24] Yevtushenko A., Topczewska K., Zamojski P. (2023). Temperature during Repetitive Short-Term Operation of a Brake with Functionally Graded Friction Element. Materials.

[25] Jojith R., Sam M., Radhika N. (2022). Recent advances in tribological behavior of functionally graded composites: A review. Eng. Sci. Technol. Int. J..

[26] Bhandari M., Purohit K. (2022). Dynamic fracture analysis of functionally graded material structures—A critical review. Compos. Part C Open Access.

[27] Yevtushenko A., Topczewska K., Zamojski P. (2021). The Effect of Functionally Graded Materials on Temperature During Frictional Heating: Under Uniform Sliding. Materials.

[28] Topczewska K., Gerlici J., Yevtushenko A., Kuciej M., Kravchenko K. (2022). Analytical Model of the Frictional Heating in a Railway Brake Disc at Single Braking with Experimental Verification. Materials.

[29] Rahmati Nezhad Y., Asemi K., Akhlaghi M. (2011). Transient solution of temperature field in functionally graded hollow cylinder with finite length using multi layered approach. Int. J. Mech. Mater. Des..

[30] Kulchytsky-Zhyhailo R., Bajkowski A.S. (2016). Axisymmetrical problem of thermoelasticity for half-space with gradient coating. Int. J. Mech. Sci..

[31] Kiani Y., Eslami M.R. (2014). Geometrically non-linear rapid heating of temperature-dependent circular FGM plates. J. Therm. Stress..

[32] Yevtushenko A., Topczewska K., Zamojski P. (2022). The Heat Partition Ratio during Braking in a Functionally Graded Friction Couple. Materials.

[33] Yevtushenko A., Kuciej M., Topczewska K., Zamojski P. (2022). Temperature in the Friction Couple Consisting of Functionally Graded and Homogeneous Materials. Materials.

[34] Lee S.W., Jang Y.H. (2009). Frictionally excited thermoelastic instability in a thin layer of functionally graded material sliding between two half-planes. Wear.

[35] Liu J., Ke L.L., Wang Y.S. (2011). Two-dimensional thermoelastic contact problem of functionally graded materials involving frictional heating. Int. J. Solids Struct..

[36] Suresh S., Mortensen A. (1998). Fundamentals of Functionally Graded Materials.

[37] Sneddon I.N. (1972). The Use of Integral Transforms.

[38] Abramowitz M., Stegun I. (1964). Handbook of Mathematical Functions with Formulas, Graphs, and Mathematical Tables.

[39] Bateman H., Erdelyi A. (1954). Tables of Integrals Transforms.

[40] Luikov A.V. (1968). Analitycal Heat Diffusion Theory.

[41] Barber J.R., Martin-Moran C.J. (1982). Green’s functions for transient thermoelastic contact problems for the half-plane. Wear.

[42] Yevtushenko A.A., Kuciej M., Topczewska K. (2017). Analytical model for investigation of the effect of friction power on temperature in the disc brake. Adv. Mech. Eng..

[43] Özis̨ik N.M. (1993). Heat Conduction.

[44] Chichinadze A.V. (1984). Polymers in Friction Assembles of Machines and Devices: A Handbook.

[45] Mao J.J., Ke L.L., Yang J., Kitipornchai S., Wang Y.S. (2018). The coupled thermoelastic instability of FGM coatings with arbitrarily varying properties: In-plane sliding. Acta Mech..

[46] Piessens R., De Doneker-Kapenga E., Überhuber C.W., Kahaner D.K. (2012). Quadpack: A Subroutine for Automatic Integration.

